# *TP53* oncomorphic mutations predict resistance to platinum- and taxane-based standard chemotherapy in patients diagnosed with advanced serous ovarian carcinoma

**DOI:** 10.3892/ijo.2014.2747

**Published:** 2014-11-11

**Authors:** PAVLA BRACHOVA, SAMUEL R. MUETING, MATTHEW J. CARLSON, MICHAEL J. GOODHEART, ANNA M. BUTTON, SARAH L. MOTT, DONGHAI DAI, KRISTINA W. THIEL, ERIC J. DEVOR, KIMBERLY K. LESLIE

**Affiliations:** 1 Department of Obstetrics and Gynecology, University of Iowa, Iowa City, IA 52242, USA; 2 Holden Comprehensive Cancer Center and Molecular and Cellular Biology Program, University of Iowa, Iowa City, IA 52242, USA

**Keywords:** oncomorphic p53 mutation, *TP53*, gain-of-function, ovarian cancer, chemoresistance

## Abstract

Individual mutations in the tumor suppressor *TP53* alter p53 protein function. Some mutations create a non-functional protein, whereas others confer oncogenic activity, which we term ‘oncomorphic’. Since mutations in *TP53* occur in nearly all ovarian tumors, the objective of this study was to determine the relationship of oncomorphic *TP53* mutations with patient outcomes in advanced serous ovarian cancer patients. Clinical and molecular data from 264 high-grade serous ovarian cancer patients uniformly treated with standard platinum- and taxane-based adjuvant chemotherapy were downloaded from The Cancer Genome Atlas (TCGA) portal. Additionally, patient samples were obtained from the University of Iowa and individual mutations were analyzed in ovarian cancer cell lines. Mutations in the *TP53* were annotated and categorized as oncomorphic, loss of function (LOF), or unclassified. Associations between mutation types, chemoresistance, recurrence, and progression-free survival (PFS) were calculated. Oncomorphic *TP53* mutations were present in 21.3% of ovarian cancers in the TCGA dataset. Patients with oncomorphic *TP53* mutations demonstrated significantly worse PFS, a 60% higher risk of recurrence (HR=1.60, 95% confidence intervals 1.09, 2.33, p=0.015), and higher rates of platinum resistance (χ^2^ test p=0.0024) when compared with single nucleotide mutations not categorized as oncomorphic. Furthermore, tumors containing oncomorphic *TP53* mutations displayed unique protein expression profiles, and some mutations conferred increased clonogenic capacity in ovarian cancer cell models. Our study reveals that oncomorphic *TP53* mutations are associated with worse patient outcome. These data suggest that future studies should take into consideration the functional consequences of *TP53* mutations when determining treatment options.

## Introduction

Epithelial ovarian cancer is the most deadly of the gynecologic malignancies and the fifth leading cause of cancer-related death among women (1). Although there has been an improvement in the 5-year survival of patients diagnosed with advanced disease, the long-term survival rate remains poor at 30% (1). Low survival can be attributed to the insidious nature of ovarian cancer progression, resulting in late diagnosis. Unfortunately, 75% of cases involve metastases to the abdominal cavity (FIGO stages III–IV) at the time of diagnosis (2). An additional complication contributing to low survival is the high rate of chemoresistance (1). The ability to predict the patients at highest risk for rapid disease progression would allow clinicians to optimize therapy up front using more aggressive regimens.

The Cancer Genome Atlas (TCGA) has provided key insight into molecular alterations that are common in ovarian tumors (3). Of note, mutations in a single gene, *TP53*, were identified in 96% of all serous ovarian tumors (3). *TP53* encodes the tumor suppressor protein p53, which acts as the major control center in the cellular response to various stress such as DNA-damaging chemotherapy. Once activated in response to chemotherapy, p53 enhances cell cycle arrest and DNA damage repair, or induces apoptosis and senescence if cellular repair is not possible.

Although almost all serous ovarian cancer patients harbor mutations in *TP53*, the mutations are extremely heterogeneous and occur at almost every codon in the DNA-binding domain of the gene (4). However, the specific *TP53* mutation can drastically alter the function of the mutated protein in a myriad of different ways. For example, studies using biochemical assays, cell models, as well as mouse and rat models have demonstrated that some *TP53* mutations abolish the wild-type (WT) function of p53 as well as confer new oncogenic activities (5). We have termed these types of mutations oncomorphic *TP53* mutations (6). Studies in cultured cancer cell lines and animal models of cancer demonstrate that oncomorphic *TP53* mutations can contribute to chemoresistance and cancer progression. However, the phenomenon has not yet been convincingly demonstrated in patients, partly due to the lack of a study population size with sufficient power to observe significant associations (7). This type of analysis is now achievable through the TCGA with the availability of clinical and genetic data from hundreds of ovarian cancer patients. Using these data, as well as findings from patients at the University of Iowa, we sought to test our hypothesis that oncomorphic *TP53* mutations in advanced serous ovarian tumors are associated with worse outcomes.

Using stringent criteria to define oncomorphic *TP53* mutations, we evaluated the relationship of oncomorphic p53 expression with progression-free survival (PFS), risk of recurrence, and response to standard platinum and taxane chemotherapy. Our data provide the first evidence that ovarian cancer patients with oncomorphic *TP53* mutations have worse clinical outcomes compared to patients with unclassified *TP53* mutations, including a shorter PFS and a 60% greater risk of recurrence. These findings have important potential implications for all cancers characterized by mutations in *TP53*.

## Materials and methods

### Ovarian cancer cell cultures

Eleven ovarian cancer cell lines were utilized in these studies. ES-2, and SKOV3 cells were cultured as monolayers in McCoy’s 5A medium. Caov3 cells were maintained in Dulbecco’s Modified Eagle’s Medium (DMEM). Ovcar3 and UCI-107 cells were cultured in RPMI-1640 medium. Caov4 and SW626 cells were maintained in Leibovitz’s L-15 medium. TOV112D and OV-90 cells were cultured in a 1:1 mixture of MCDB 105 medium containing 1.5 g/l of sodium bicarbonate and medium 199 containing 2.2 g/l sodium bicarbonate. UWB1.289 cells were grown in a 1:1 mixture of RPMI-1640 and Mammary Epithelial Growth Medium (MEGM) (Clonetics/Lonza). All media conditions were supplemented with 10% fetal bovine serum (FBS) and 1 U/ml penicillin and 10 μg/ml streptomycin and cells were maintained in a humidified incubator with 5% CO_2_ at 37°C. All cell lines are available from American Type Cell Culture, except UCI-107 cells that were generously gifted from Dr Michael J. Goodheart.

The cell line SKOV3 has a loss of function (LOF) *TP53* mutation that results in a lack of p53 protein expression. This cell line was used as a model to study the effects of the most common oncomorphic *TP53* mutations by stably expressing the following mutants in *TP53*: R175H, R248Q, R248Q.P72R, R248W, R273C, R273L, R273S, and Y220C as previously described (8).

### Western blot analysis

Analysis of protein expression/phosphorylation was performed as previously described (9) for the following proteins: p53 (sc-126; Santa Cruz Biotechnology, Inc.), p21 (no. 2947), ERCC1 (no. 12345), c-Myc (no. 9402), β-catenin (no. 9582), mammalian target of rapamycin (mTOR) (no. 2983) (all from Cell Signaling Technology, Inc.), p-Rb S807 (no. ab47762; Abcam), and β-actin (no. A1978; Sigma).

### Clonogenic survival

Cells were trypsinized and plated in triplicate into 60 mm tissue culture dishes at 800 cells/well. Twenty-four hours later, cells were treated with 1 μM cisplatin or 5 nM taxol for 48 h. Fresh media was added and cells were allowed to grow for 21 days. Viable clones were visualized by staining with crystal violet, and colonies >50 cells were counted. Plating efficiency was calculated by dividing the average number of colonies per plate by the number of cells plated. Surviving fractions were calculated by normalization to the plating efficiency.

### Subjects

**Figure 1:**
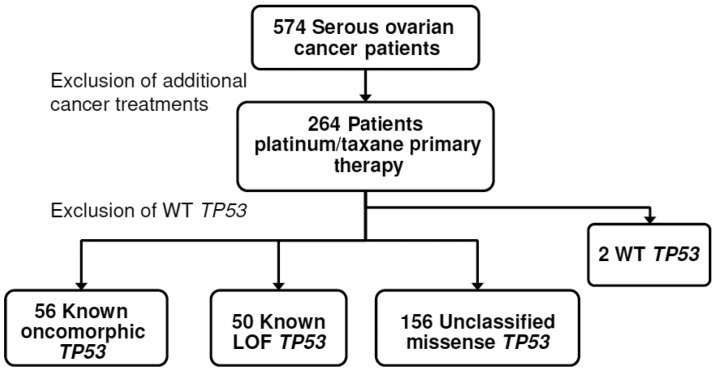
Inclusion criteria to study the effect of *TP53* mutation type on serous ovarian cancer patient outcomes. Out of 574 serous ovarian cancer patients included in The Cancer Genome Atlas (TCGA) dataset, patients included in our study had uniform platinum- and taxane-based primary chemotherapy. Of those, we determined the *TP53* mutation based on exon sequencing and discriminated each mutation into four groups: known oncomorphic, known loss of function (LOF), unclassified, or wild-type (WT). Oncomorphic mutations were designated based on *in vivo* or *in vitro* evidence of an oncogenic phenotype. LOF mutations contain a nonsense or a frameshift mutation. Remaining *TP53* mutations are designated as unclassified mutations, and have unknown functions.

Clinical, genetic, and protein expression data from 264 advanced serous ovarian cancer patients without a previous cancer history were downloaded from the TCGA data portal (accessed 05/06/2013). Analyses were limited to data from those patients who received platinum (carboplatin, cisplatin, or oxaliplatin)- and taxane (Taxotere or Paclitaxel)-based chemotherapy (Fig. 1). Clinical characteristics of the study cohort are listed in Table I. An independent validation patient cohort (n=32) was obtained from the University of Iowa Gynecologic Oncology Tumor Bank. The University of Iowa Institutional Review Board approved these studies. The same inclusion criteria were used for both patient cohorts: patients were of advanced stage (III or IV), specific *TP53* sequencing information was available, and clinical outcome was known.

### Criteria for designating TP53 mutations

*TP53* mutations were binned into three categories: oncomorphic, LOF, and unclassified. Oncomorphic mutations were designated based on previously published studies showing that a particular mutation causes an oncogenic phenotype. For example, Hanel *et al* used a knock-in mouse to determine the function of two common mutations (10). Compared with the p53 null mouse (p53^−/−^), a mouse carrying a p53 R248Q allele (p53^R248Q/−^) displayed accelerated tumor onset and shortened survival, but a mouse model carrying a p53 G245S allele (p53^G245S/−^) showed no differences in survival when compared with the p53^−/−^ mouse (10). These are some of the first data indicating that *TP53* mutations vary in function with respect to tumorigenicity. Eight *TP53* mutations were considered oncomorphic, and were selected based on previous *in vivo* and *in vitro* studies [P151S (11,12), Y163C (13), R175H (14–16), L194R (17), Y220C (18), R248Q (10), R248W (19,20), R273C (21,22), R273H (15,19,23), R273L (24), R282W (13)]. LOF mutations were defined as i) point mutations that create a stop codon (nonsense mutation); or ii) frame shift mutations that cause significant disruptions in the translation of the protein. WT mutations were defined as mutations that do not alter the amino acid sequence. The remaining mutations were single nucleotide substitutions, the function of which is not fully known at this time, but do not meet oncomorphic criteria. These were categorized as ‘unclassified’ mutations. Splice mutations located at the intron-exon borders were categorized into the ‘unclassified’ category due to conflicting studies on their function (25–28).

### Defining clinical endpoints

Clinical details available from the TCGA portal were used to document the following clinical endpoints: PFS and platinum status. PFS was defined as the interval between the date of initial surgical removal of the tumor to the date of progression in patients who were not cancer free, or date of recurrence. Chemotherapy details were available that documented the date of last primary platinum treatment. Platinum-free interval was defined as the interval between last primary platinum treatment to the date of progression or recurrence. Platinum status was defined as resistant if the platinum-free interval was <6 months when the patient recurred. Platinum status was defined as sensitive if the interval to recurrence was >6 months, or the follow-up period for those lost to contact was >6 months from the date of the last platinum treatment. Patients who did not progress or have a recurrence were censored in both analyses at the date of the last-known contact.

### RPPA protein data

Corrected and normalized reverse phase protein array (RPPA) data were downloaded from the TCGA portal to analyze protein expression differences between patients with oncomorphic, LOF, or unclassified mutations. Detailed information on normalization has been previously reported (3); briefly, the raw data were converted from a log 2 value into an arbitrary linear value and corrected based on the normalization of means among all patient samples.

### Statistical analysis

To determine if different mutations confer worse patient outcome, plots of the Kaplan-Meier estimated cumulative probabilities of PFS were constructed. Cox proportional hazard regression was utilized to test for differences in PFS between mutation types using a study endpoint of 60 months, as previously reported (4). To assess for group differences between the mutations on relevant clinical variables, a χ^2^ test or Fisher’s exact test was utilized where appropriate. A Kruskal-Wallis or Wilcoxon rank sum test was performed to detect differential protein expression between all three mutation groups, or between two groups, respectively. All tests were two sided and tested at the 5% significance level. The data analysis was generated using SAS software, version 9.3 (SAS Institute, Inc.).

## Results

### Selection of patient population

As shown in Fig. 1, the primary exclusion criterion was patient exposure to treatment beyond adjuvant primary chemotherapy with platinum and taxane. The median PFS for the study population was 13.8 months, and median overall survival was 30.2 months, which is consistent with reported outcomes in the full TCGA ovarian cancer data set (3).

### Frequency and spectrum of TP53 mutations

Exon sequencing data were downloaded from the TCGA portal and mutations in *TP53* were annotated. Two patients had synonymous missense mutations that retained the integrity of WT p53 protein sequence and were designated as WT. Data for these two patients were excluded due to insufficient sample size (Fig. 1).

**Figure 2:**
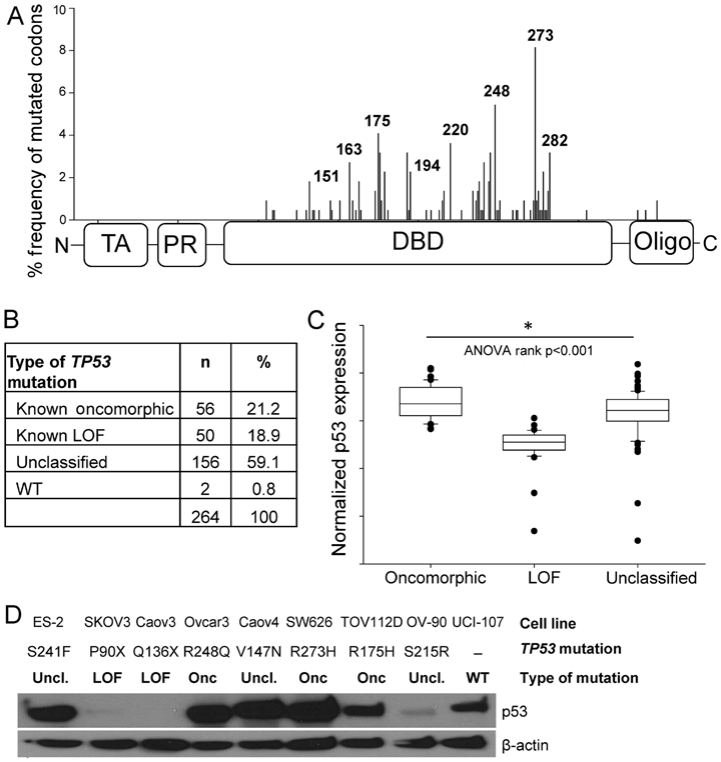
Landscape of *TP53* mutations in the study population. (A) *TP53* gene structure and frequency of *TP53* mutations at individual codons in patients included in this study. Denoted codons are oncomorphic alterations. (B) Number and frequency of *TP53* mutations in study cohort categorized by functional consequence. (C) Normalized protein expression of p53 in serous ovarian cancer tumors in the three functional categories of *TP53* mutations. ^*^Kruskal-Wallis tests were performed to assess significance. (D) Baseline p53 expression in a panel of nine ovarian cancer cell lines. TA, transactivation domain; PR, proline rich domain; DBD, DNA-binding domain; Oligo, oligomerization domain; LOF, loss of function; Uncl., unclassified.

Mutations in *TP53* occurred predominately in the DNA-binding domain (Fig. 2A), consistent with a previous report (4). The most common mutations occurred at codons R273 (6.1%), R248 (4.6%), and R175 (3.4%). Oncomorphic mutations comprised 21.2% of the patient population, LOF mutations comprised 18.9%, and the remaining 59.1% were unclassified mutations (Fig. 2B). Splice mutations located at the intron-exon borders were categorized as ‘unclassified’ due to conflicting studies on their function (25–28). Splice mutations occurred in 10% of our study population, a frequency much larger than previously reported (27). We speculate that the advanced technology used to sequence *TP53* exons is more sensitive than used previously. The frequency of oncomorphic and LOF mutations in this cohort is similar to that calculated from the International Agency for Research on Cancer p53 database (4,6), thus validating our study population.

To confirm our classification of oncomorphic and LOF mutations, we analyzed normalized protein expression of p53 as reported in the RPPA data set. LOF mutations result in loss of p53 protein expression, whereas oncomorphic p53 has been reported to be hyper-stabilized (5). As expected, we detected a significant difference in protein levels of p53 for the oncomorphic, LOF and unclassified mutations (Fig. 2C, p<0.001). Specifically, tumors containing oncomorphic *TP53* mutations had the highest p53 protein levels, whereas tumors with LOF *TP53* mutations displayed the lowest expression of p53. Tumors with unclassified mutations had a broad range of p53 protein expression.

We utilized a panel of nine ovarian cancer cell lines with various *TP53* mutations to characterize expression levels of mutated p53 proteins (Fig. 2D). Three cell lines with oncomorphic *TP53* mutations displayed abundant mutated p53 protein expression. Two cell lines with LOF *TP53* mutations did not express p53 protein; and cell lines with unclassified *TP53* mutations demonstrated a range of p53 protein expression. One cell line, UCI-107, expresses WT *TP53*.

### Oncomorphic mutations in TP53 confer worse patient outcome

**Figure 3:**
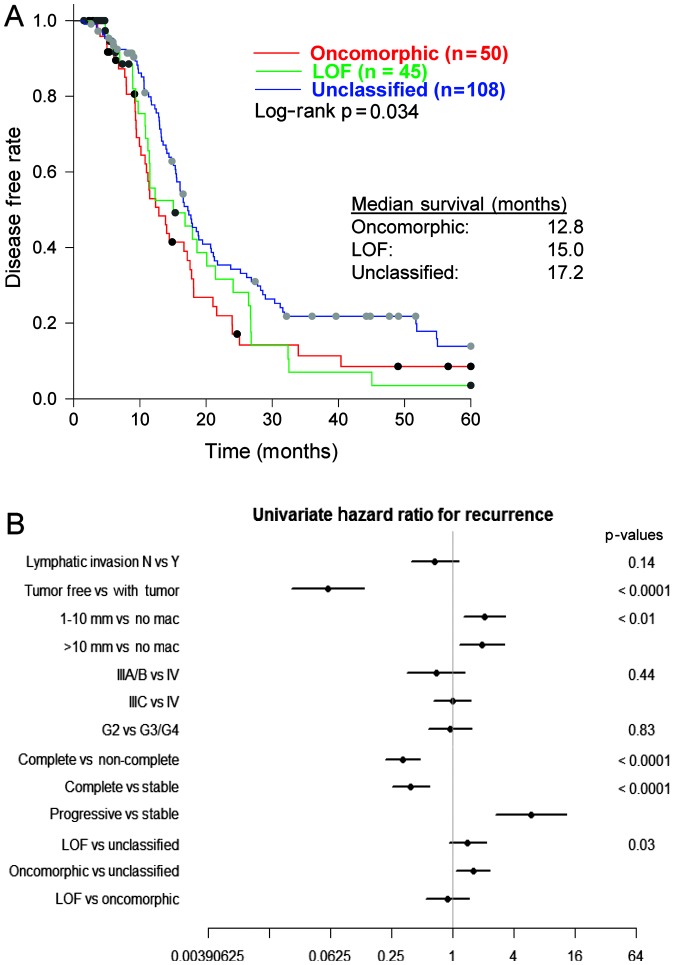
Oncomorphic *TP53* mutations are associated with worse progression-free survival (PFS) and increased risk of recurrence. (A) Plot of PFS. Log-rank test was used to assess significance among the three *TP53* mutational categories. Median PFS is noted in inset. (B) Hazard ratio plot showing clinical factors associated with recurrence. No mac, no macroscopic disease; G, grade; LOF, loss of function.

**Figure 4:**
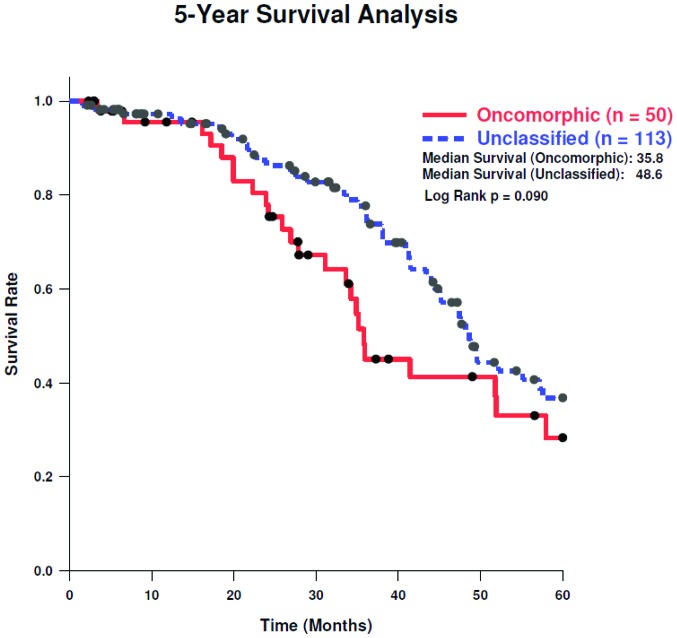
Five-year overall survival rate in patients with oncomorphic and unclassified *TP53* mutations. Plots of the Kaplan-Meier estimated cumulative probabilities of overall survival were constructed. Cox proportional hazards regression was utilized to test for differences in progression-free survival (PFS) between mutation types using a study endpoint of 60 months.

We assessed the association of oncomorphic *TP53* mutations with patient outcome, by first calculating PFS among patients with oncomorphic, LOF, or unclassified mutations and found a significant difference between categories (p=0.03). Follow-up pairwise comparisons demonstrated that patients with oncomorphic *TP53* mutations showed significantly worse PFS when compared with patients harboring unclassified mutations (p=0.015) (Fig. 3A). The median PFS was 12.8, 15.0, and 17.2 months for patients with oncomorphic, LOF, and unclassified mutations, respectively. Analysis of 5-year survival revealed a trend towards better survival in patients with unclassified mutations as compared to oncomorphic mutations (Fig. 4, log-rank test p=0.11).

To provide further insight into which clinical factors may be contributing to the differing PFS outcomes between mutational classifications, a univariate comparison of clinical factors was conducted (Table II). Patients with oncomorphic *TP53* mutations displayed higher rates of platinum resistance when compared with LOF and unclassified mutants (χ^2^ test p=0.0024). More than half (51.2%) of patients with oncomorphic mutations displayed platinum resistance, whereas patients with unclassified mutations had the highest rates of platinum sensitivity (Table II). In addition, patients with oncomorphic *TP53* mutations had almost 60% higher odds of recurrence (HR=1.60, 95% confidence intervals 1.09, 2.33, p=0.015) when compared to patients with other unclassified mutations (Fig. 3B). We also observed the anticipated associations of recurrence with residual disease and response to therapy (Fig. 3B).

**Figure 5:**
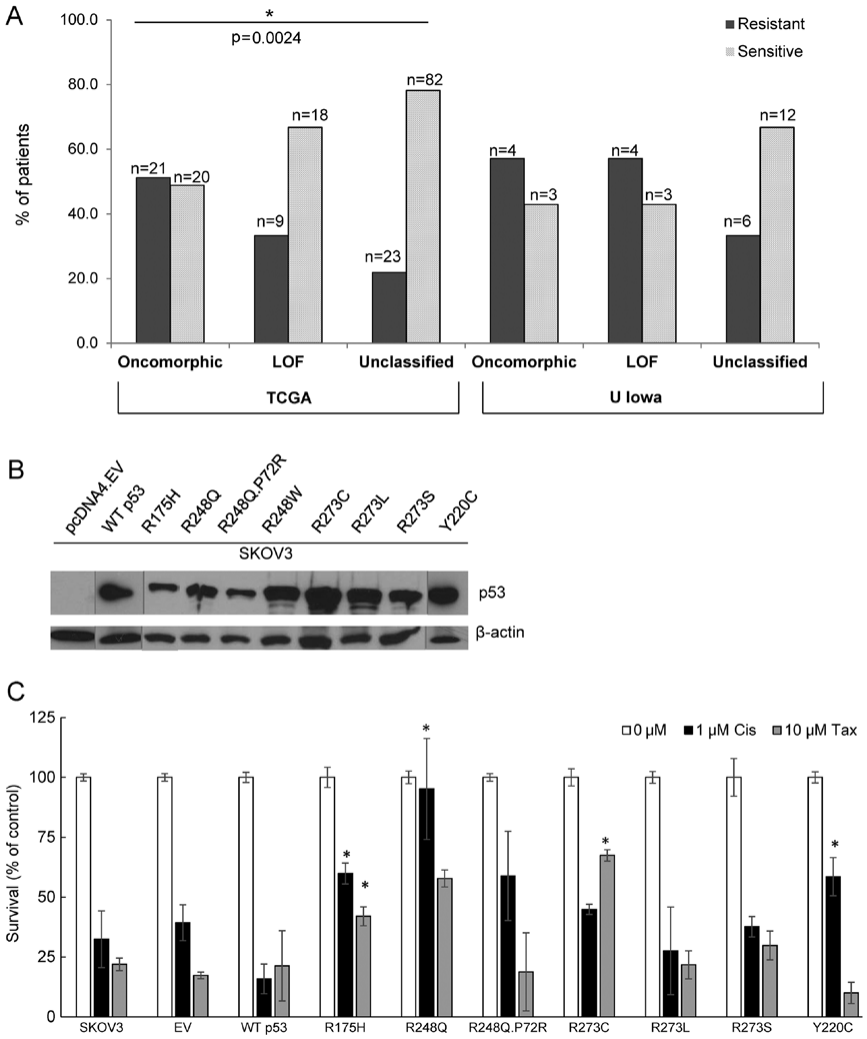
Tumors with oncomorphic *TP53* are more resistant to chemotherapy than patients with loss of function (LOF) or unclassified mutations. Rates of platinum resistance among The Cancer Genome Atlas (TCGA) cohort and a validation cohort from the University of Iowa. Number of patients (n) are noted in the plot. (B) The most common *TP53* mutations were expressed in a cell line (SKOV3, p53 null) to examine the ability of oncomorphic p53 mutant proteins to cause chemoresistance to cisplatin or taxol. Western blot images are combined from two separate gels (demarked by the gray lines separating lanes from different gels). (C) Clonogenic survival of cells stably expressing various *TP53* mutant proteins after 48 h treatment with 1 μM cisplatin (Cis) or 10 nM taxol (Tax). ^*^P<0.05 vs. empty vector (EV) with the same treatment.

To validate the clinical and genetic data obtained from the TCGA, we determined rates of chemoresistance in patients who were diagnosed with ovarian cancer and had banked tumors at the University of Iowa. Sequencing information on *TP53* was available for all tumors. We observed a similar trend towards resistance in tumors with oncomorphic *TP53* (Fig. 5A). In addition, patients with unclassified *TP53* mutations demonstrated the highest sensitivity to chemotherapy. A p53 null cell line (SKOV3) was utilized to express the most common *TP53* oncomorphic mutations (Fig. 5B). Clonogenic survival in response to cisplatin treatment was enhanced by cells expressing R175H, R248Q, and Y220C oncomorphic p53 mutant proteins. In response to taxol chemotherapy, clonogenic survival was enhanced in cells expressing the R175H and R273C p53 mutated proteins (Fig. 5C).

### Protein expression differences between oncomorphic mutations and unclassified mutations

**Figure 6:**
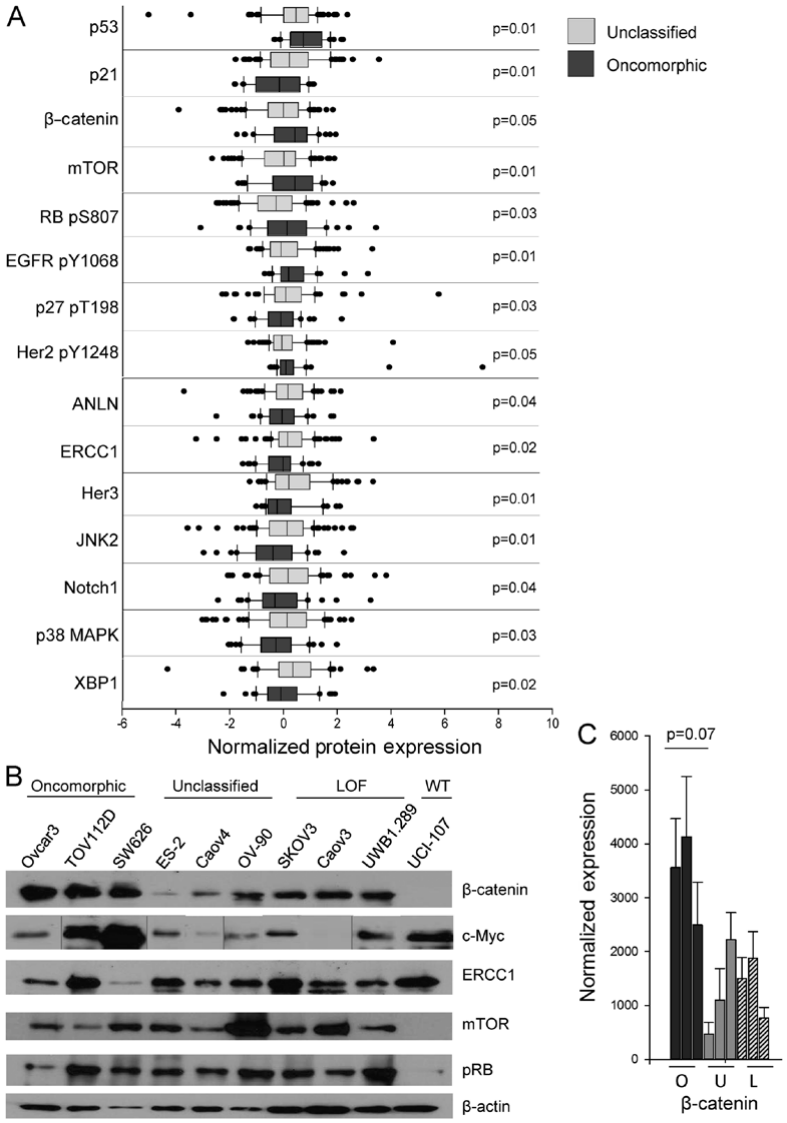
Tumors with oncomorphic *TP53* mutations have elevated expression and activity of proteins involved in tumor growth as compared to tumors with unclassified mutations. (A) Reverse phase protein arrays (RPPAs) were used to determine protein expression in The Cancer Genome Atlas (TCGA) analysis. Normalized protein expression was downloaded and compared using a Wilcoxon rank sum test to identify proteins differentially expressed between the two groups. The full dataset is available in Table III. Comparison of RPPA data among oncomorphic, loss of function (LOF), and unclassified *TP53* are available in Table IV. (B) Analysis of the expression of proteins identified through the TCGA analysis in a panel of ovarian cancer cell lines. Western blot image of c-Myc contains gray lines demarking re-arrangement of the image, however it is an image of the same blot. (C) Densitometry analysis of β-catenin trended towards higher expression in the panel of cell lines, tested by Kruskal-Wallis test p=0.07.

We next interrogated possible mechanisms of chemoresistance in tumors containing oncomorphic mutations by comparing protein expression profiles between oncomorphic and unclassified mutations. Data, which are part of TCGA dataset, were obtained by RPPA, a high-throughput technique for simultaneous measurement of protein expression in a large number of biological samples using antibody-based methods. We observed differential expression of 15 different proteins in tumors with either oncomorphic or unclassified *TP53* mutations (Fig. 6A, Table III). In particular, the pro-apoptotic protein BAK and the cell cycle regulator p21 (CIP1/WAF1) were expressed at a significantly lower level in tumors with oncomorphic *TP53* mutations. β-catenin, phosphorylated epidermal growth factor receptor (EGFR) (Y1068), and mTOR were significantly elevated in patients with oncomorphic *TP53* mutations compared with patients with unclassified mutations. Further evaluation of the RPPA data from the three mutational categories (oncomorphic, LOF and unclassified) revealed differences in tumor protein expression (Table IV). To further define the most significantly altered pathways in cells with oncomorphic *TP53*, we assessed the expression of the targets identified from the TCGA dataset in representative cell lines (Fig. 6B). The most highly correlated alterations were in the β-catenin pathway, known to be associated with ovarian cancer carcinogenesis and proliferation (29) (Fig. 6C).

## Discussion

Recent advances in cancer biology involve understanding the effects of mutations in *TP53* on the function of the mutant protein (5). Many clinical studies have attempted to correlate the presence of a *TP53* mutation with patient survival or the development of chemoresistance (7). The results of these studies are conflicting primarily because of indiscriminate grouping of *TP53* mutations with different functions (oncomorphic, LOF and unclassified). Given that 21% of all ovarian cancer patients harbor oncomorphic *TP53* mutations, studies which take into account the functional implications of these mutations are vital. The availability of a large cohort of ovarian cancer tumors and corresponding clinical data through TCGA has made it possible to address the clinical consequence of oncomorphic mutations in *TP53* for the first time and to confirm the mechanistic implications of oncomorphic p53 expression in representative cell models. Thus, the objective of our study was to determine if oncomorphic *TP53* mutations are associated with worse patient outcomes. We demonstrate that oncomorphic *TP53* mutations predict for worse PFS and higher rates of chemoresistance and recurrence. Preclinical models confirm the oncomorphic function of the identified *TP53* mutations and suggest mechanisms by which oncomorphic *TP53* drive ovarian cancer cell growth.

Although sequence similarities exist among many p53 mutant proteins, to date only stringent biological, *in vivo* assays can determine oncomorphic properties (6). Accordingly, a previous study using less stringent criteria to define ‘gain of function’ *TP53* mutations did not find a significant relationship between the gain of function mutations and chemoresistance (30). Herein we used more stringent criteria to define oncomorphic mutations and propose that our findings more clearly delineate the impact of these oncogenic proteins. Our criteria required that mutations increase clonogenic potential *in vitro* or increase tumorigenesis *in vivo* as compared to *TP53*-null mice to be considered oncomorphic (10–24,31,32). Using these criteria, we found that the presence of a *TP53* oncomorphic mutation in a patient tumor specimen predicts for platinum resistance.

To understand the oncogenic properties of oncomorphic p53 proteins, we analyzed differential protein expression between the *TP53* mutation groups. The cell cycle regulator p21, which is induced by p53 and results in cell cycle arrest, was expressed at a low level in tumors containing oncomorphic *TP53* mutations. Levels of phosphorylated p27 were also lower in these samples. Conversely, tumors with unclassified *TP53* mutations displayed higher p21 expression, suggesting that some of the unclassified mutations may have residual WT p53 functions. Previous studies have demonstrated that positive p21 staining in ovarian tumor specimens correlates with an overall survival advantage (33,34). Our data also indicated that tumors with oncomorphic *TP53* have increased expression of activated pro-growth pathways, such as phosphorylation of EGFR, Her2, and retinoblastoma protein (Rb). EGFR phosphorylation at Y1068 is a hallmark for activated EGFR signaling and is the site of Grb2 and Ras binding that perpetuate Ras activation and mitogen-activated protein kinase signaling (35). The proteins mTOR and β-catenin, which are commonly overexpressed in cancer, were also significantly increased in oncomorphic *TP53* tumors, indicating enhanced pro-survival signaling, however. Recently, high β-catenin was associated with poor ovarian cancer patient outcome (29). This protein was the most highly altered in our panel of ovarian cancer cell lines as well as in patient tumors. These data correlate well with *in vitro* studies showing that EGFR is a direct transcriptional target of oncomorphic p53 proteins (36). In addition, others have shown that oncomorphic p53 regulates expression of key cell cycle regulators (37). Understanding the molecular consequences of oncomorphic *TP53* mutations has the potential to identify key signaling targets that could be blocked in order to overcome chemoresistance in tumors with these oncogenic mutations.

Patients whose tumors expressed unclassified *TP53* mutations made up the majority of the ovarian cancer study population. These patients represent an interesting clinical population since our data demonstrate that patients harboring unclassified mutations are significantly more sensitive to chemotherapy and have lower rates of recurrence. Tumors with unclassified *TP53* mutations express the mutated p53 protein at a fairly high level, and it is possible that these proteins have some residual WT p53 function as evidenced by higher expression of p21. The overall survival of patients with unclassified mutations trended towards improved 5-year survival as compared to oncomorphic mutations. Note, however, that overall survival data are not mature for some patients in TCGA dataset; thus, overall survival should be re-examined when these data are complete.

Although two patients with WT *TP53* were excluded from our study, and these patients are rare in advanced ovarian cancers, a recent study of 11 ovarian tumors with WT p53 reported a worse overall survival and PFS as compared to a mutated *TP53* (38). The study by Wong *et al* represents a step towards understanding how p53 function affects outcomes, but it remains unclear why the tumors with functional p53 fail to respond to standard DNA-damaging chemotherapy (38). One possibility is that other mutations present in the tumors drive drug resistance; another possibility is that WT p53 enforces cell cycle checkpoints, making the cells less vulnerable to chemotherapeutic agents which act specifically in mitosis (9).

An important aspect of p53 biology is the integrity of the second *TP53* allele. Mutant p53 proteins can exert dominant negative activity by inhibiting DNA binding and hence, the tumor-suppressive function of the remaining WT *TP53* allele (39). The status of both alleles is necessary to have a complete understanding of the effect of a particular mutation; however this is a limitation of the TCGA data. The use of exon sequencing did not distinguish between loss of heterozygosity (LOH) or tumor heterogeneity (3). Future studies will need to take this into account.

In conclusion, almost all advanced serous ovarian tumors contain *TP53* mutations. Understanding the p53 mutational category, which significantly impacts function, is critical to predicting patient outcomes. Specifically, we demonstrate that patients with oncomorphic *TP53* mutations are significantly more resistant to chemotherapy, have shorter PFS and a higher risk of recurrence. A recent study in Li-Fraumeni syndrome patients analyzed the individual impact of common *TP53* missense mutations and identified a particular mutation (R282W) that results in earlier onset of tumor formation (40). Such patients, and patients identified in our study with oncomorphic *TP53* mutations deserve careful follow-up post-therapy and may require novel treatment regimens to improve outcomes. In addition, when studying the impact of new therapies in ovarian cancer, we propose that stratification should be considered based upon p53 mutational category.

## Figures and Tables

**Table I:** Clinical and pathological characteristics of TCGA serous ovarian tumors from patients treated with standard platinum- and taxane-based chemotherapy.

Characteristic	n	%
Age at diagnosis		
<60 years	148	56.06
≥60 years	116	43.94
Vital Status		
Dead	126	47.73
Alive	138	52.27
Tumor grade		
G2	21	7.95
G3/G4	236	89.39
Unknown	7	2.65
FIGO stage		
IIIA/B	21	7.95
IIIC	197	74.62
IV	46	17.42
Lymph invasion		
No	38	14.39
Yes	63	23.86
Unknown	163	61.74
Residual disease		
≤1 cm	126	47.73
>1 cm	60	22.73
Complete removal	51	19.32
Unknown	27	10.23
Clinical response to chemotherapy		
Complete response	155	58.71
Partial response	24	9.09
Stable disease	19	7.20
Progressive disease	12	4.55
No data	54	20.45
Platinum status		
Resistant	49	20.25
Sensitive	112	46.28
Too early	34	14.05
Unknown	47	19.42
p53 mutation type		
LOF	51	19.32
Oncomorphic	56	21.21
Unclassified	154	58.33
WT	2	0.76
Unknown (no sequence information available)	1	0.38

TCGA, The Cancer Genome Atlas; LOF, loss of function; WT, wild-type.

**Table II:** Univariate analysis of association of clinical factors with *TP53* mutation categories (oncomorphic, LOF, and unclassified) demonstrates that platinum status is significantly different among the three mutation groups.

Variable	Category	n			p-value χ^2^ test

		Oncomorphic	LOF	Unclassified	
Lymphatic invasion	No	13	6	19	0.0767
	Yes	9	13	39	
Tumor grade	G2	4	5	12	0.8373
	G3/G4	50	43	138	
Cancer status	Tumor free	15	12	45	0.8439
	With tumor	37	33	100	
Residual tumor	≤1 cm	28	26	70	0.5075
	>1 cm	11	13	33	
	No mac	15	6	30	
Tumor stage	IIIA/B	6	4	11	0.8529
	IIIC	40	36	117	
	IV	10	10	25	
Vital status	Dead	28	22	73	0.8234
	Alive	28	28	80	
Platinum status	Resistant	21	9	23	0.0024
	Sensitive	20	18	82	
Therapy outcome	Complete response	33	29	76	0.0970
	Progressive disease	4	2	4	
	Stable disease	4	14	20	

LOF, loss of function; mac, macroscopic disease.

**Table III:** Significant differential protein expression between tumors with oncomorphic versus unclassified *TP53* mutations as determined by Wilcoxon rank sum test. Median protein expression is presented.

Analysis variable	Median expression		p-value Wilcoxon test (two sided)

	Oncomorphic	Unclassified	
ANLN	−0.08	0.14	0.042568565
β-catenin	0.40	−0.03	0.047657921
EGFR pY1068	0.16	−0.12	0.007949216
ERCC1	−0.04	0.12	0.015114876
Her2 pY1248	0.06	−0.08	0.049131573
Her3	−0.26	0.17	0.010622594
JNK2	−0.42	0.10	0.00516184
mTOR	0.40	−0.01	0.008801626
Notch1	−0.35	0.14	0.040835561
p21	−0.18	0.19	0.007848037
p27 pT198	−0.13	0.05	0.034859604
p38 MAPK	−0.31	0.10	0.025973549
p53	0.71	0.43	0.008155086
RB pS807 S811	0.11	−0.30	0.028065342
XBP1	−0.13	0.32	0.020961678

EGFR, epidermal growth factor receptor.

**Table IV:** Significant differential protein expression among tumors with oncomorphic, LOF and unclassified *TP53* mutations as determined by Kruskal-Wallis test. Median protein expression is presented.

Analysis variable	Median expression			p-value Kruskal-Wallis test

	Oncomorphic	LOF	Unclassified	
ANLN	−0.08	−0.09	0.14	0.044578193
BAX	−0.11	0.12	−0.13	0.02924444
Beclin	0.01	−0.24	0.12	0.047399782
CD31	0.15	−0.23	0.12	0.019310324
CMET pY1235	−0.16	−0.26	0.09	0.040573763
EGFR pY1068	0.16	−0.21	−0.12	0.025684237
Her3	−0.26	−0.23	0.17	0.003727584
JNK2	−0.42	0.26	0.10	0.002990478
mTOR	0.40	0.07	−0.01	0.030649059
p21	−0.18	−0.20	0.19	0.027093184
p53	0.71	−0.90	0.43	4.47E-16
PCNA	0.01	0.73	−0.13	0.009614359
RBM3	−0.09	0.60	0.08	0.018983458
RB pS807 S811	0.12	0.11	−0.30	0.022229704
XBP1	−0.13	−0.18	0.32	0.000881485

LOF, loss of function.
